# Pediatric oncology in India: Past, present and future

**DOI:** 10.4103/0971-5851.65333

**Published:** 2009

**Authors:** Brijesh Arora, Sripad D. Banavali

**Affiliations:** *Editor, IJMPO, Associate Professor, Division of Pediatric Oncology, Department of Medical Oncology, Tata Memorial Hospital, Parel, Mumbai - 400 012, India E-mail: editor@ijmpo.org*; 1 *Professor and Head, Department of Medical Oncology, Tata Memorial Hospital, Parel, Mumbai - 400 012, India*

India, in many ways, is shining today. It has transformed in to an economic powerhouse and is slated to become the third largest economy in the world. It has taken giant strides toward alleviating poverty and eliminating caste and gender inequality. Sadly, this economic progress has not percolated down to the other domains, including medical care. For us, concerned with the care of children with cancer, the state of pediatric oncology in the country today leaves a lot to be desired.

Progress in Pediatric Oncology is one of the biggest success stories in oncology in the last millennium. The 5-year survival for all pediatric cancers is now 75–80%.[1] However, the outlook of pediatric oncology in most resource-challenged countries including India is appalling.[2] Although we have made steady progress in the last few decades, we are still far behind the current international standards. It is therefore pertinent and timely to introspect and analyze the past, take stock of the current challenges and opportunities and use these insights to plan the future. Pediatric oncology in India lags behind the West in all three domains: service, research and education. It is the systematic and all-round development in all these domains which will propel pediatric oncology in India to among the best in the world.

## Pediatric oncology service

Pediatric oncology as a specialty was virtually nonexistent in the early 1980s in India. Most children were treated, often unsuccessfully, by adult oncologists in a few cancer centers or by self-trained pediatricians in medical colleges. There was lack of good quality pediatric cancer units (PCU) and multidisciplinary or protocol based care. There were only a handful of pediatric oncologists, who were usually trained abroad. The first dedicated pediatric cancer unit was started in Tata Memorial Hospital in 1985.[3] In a nationwide survey of pediatric oncology services in 1988, 50% of cancer centers had adult oncologists treating children, only 10% had trained pediatric oncologists, and less than 15% had dedicated beds for pediatric patients or facilities for platelet transfusion.[4] A similar survey done recently in more than 275 medical colleges or cancer centers revealed a better but far from ideal picture – more than 50% of medical colleges did not have facilities or expertise for treating children with cancer.[5] Even in the treating centers, availability of facilities like immunophenotyping, nuclear imaging, cytogenetics, trained oncology nurses, nutritionists, radiotherapy, blood components and morphine is very limited. Apart from poor infrastructure and lack of trained staff, sociocultural and economic factors such as limited financial resources, ignorance and cancer illiteracy contribute to advanced presentation and poor outcome of childhood cancers in India.[6] Despite these issues, the outcome of pediatric cancers has gradually improved in the country over the last four decades. The outcome of hematological cancers in terms of long-term survival has greatly improved from 20% to 60% in acute lymphoblastic leukemia (ALL), from <70% to more than 90% in Hodgkin’s disease, from 30% to 70% in non-Hodgkin’s lymphoma (NHL) and from 10% to 40% in acute myeloblastic leukemia (AML).[7–13] Similarly, the outcome in solid tumors has also improved. However, the outcome is still considerably poor compared to western figures, especially in tumors like retinoblastoma, leukemias, CNS tumors and germ cell tumors. Furthermore, there is considerable disparity in outcomes between centers with only a few achieving outcome comparable to the west.[6,11–13]

## Pediatric oncology research

The foundation of pediatric oncology research is accurate knowledge of epidemiology of childhood cancers in the country. Unfortunately, there is a real paucity of epidemiologic data on pediatric cancers in India. There was virtually no data on incidence or distribution of pediatric cancers until 1982, when the National Cancer Registry Program (NCRP) was started. From six registries and coverage of less than 2% of the population, the NCRP has expanded to 18 registries and more than 5% coverage.[14] However, the data on pediatric cancers are still limited and show great variation between different populations in India. The age-standardized rates of childhood cancers are highest (108 per million) in metropolitan areas, followed by other urban (86 per million) and rural (53 per million) areas in India. Apart from real differences in incidence, these numbers also likely reflect other factors like underdiagnosis and underascertainment in semiurban or rural areas with limited availability of diagnostic and clinical facilities.[15] To complement these efforts, a non-governmental organization called “JivDaya Foundation” has recently launched the Indian Pediatric Oncology Initiative™, with free access to an online web-based India Pediatric Oncology Database (IndiaPOD) and training for data managers at various PCUs. This would help create many new PCU-registries, which will decrease the underascertainment of cases. Furthermore, prospective data collection regarding patterns of care and outcome at each PCU would provide individual institutions with the ability to incrementally improve care and serve as a platform for collaborative studies in the future.[16]

Our country lacks a culture of organized clinical research exemplified by virtual absence of good prospective published studies on epidemiology, biology or outcome of childhood cancers. Of late, this trend is changing with some good publications. The benefits of arsenic in pediatric APML and high-dose cytarabine in T- acute lymphoblastic leukemia (T-ALL) are examples of important clinical observations by Indian centers.[17,18]

Worldwide, the outcome of pediatric cancers has improved through adoption of uniform guidelines and systematic enrolment of patients on prospective multicentric clinical trials conducted by national cooperative groups.[19] However, India lacks a national pediatric oncology research group and less than 15% patients are enrolled on clinical trials compared to more than 90% in the west.[5,20] An important effort at multicenter collaboration was the one between the US National Cancer Institute and three Indian centers, which led to the development of the MCP-841 protocol for pediatric ALL. This protocol has been one of the most important success stories of the Indian pediatric oncology community with long-term survival in pediatric ALL improving from 20 to 60% with the widespread adoption of MCP-841. This was possible through the use of a uniform treatment regimen, incremental improvement in the protocol with local input, well-organized data collection and access to experts.[6,8] We believe that there are important lessons learnt from the MCP-841 experience that would serve us well as we move forward.

## Pediatric oncology education

The pediatric oncology education has three key target groups and goals:

1. Provide formal pediatric hematology–oncology training fellowships to postgraduate students in order to create a pool of academically oriented pediatric oncologists.
2. Provide practical short-term training to interested pediatricians for shared care in satellite centers.
3. To educate primary care practitioners and pediatricians in the early diagnosis and prompt referral of childhood cancers.

There was no formal fellowship program in pediatric haematology–oncology until 2008. Recently, a few institutions have started offering fellowships or degrees under the aegis of national board or other reputed universities. The commencement of programs such as DM in Pediatric Hematology–Oncology in reputed institutions like the Post Graduate Institute of Medical Education and Research is an excellent beginning that other large centers need to emulate. For shared care, an important initiative is the National Training Program in Practical Pediatric Oncology (NTP-PPO) initiated by Pediatric Hematology Oncology (PHO) Chapter of the Indian Academy of Pediatrics (IAP) to impart practical training to interested clinicians.[21] For sensitization to early diagnosis and prompt referral, the Indian Cooperative Oncology Network has recently proposed a PromOTE-Pediatric campaign (Promotion of Oncology Training and Education in Pediatric Cancers). These efforts in furthering the cause of pediatric oncology education are at a nascent stage and would need strong and continuing support of all interested stakeholders to make a meaningful and lasting impact.

## What should be the future course of pediatric oncology in India?

The future strategy should focus on all the three aspects of pediatric oncology. To strengthen pediatric oncology service, the best way forward is through optimal utilization of existing infrastructure by creation or augmentation of PCUs at university hospitals and regional cancer centers, equipped with necessary diagnostic and therapeutic facilities. These PCUs should be linked to satellite centers with pediatricians trained in shared care. To promote pediatric oncology research, an important initial step would be the creation of a national childhood cancer registry that could generate data related to the epidemiology and end-results like the Surveillance Epidemiology and End Results (SEER) program in US. This registry could collate the prospective data gathered through nationwide usage of IndiaPOD along with NCRP. The most important step would be the establishment of a national pediatric oncology research group. A proposal by PHO to create InPOG (Indian Pediatric Oncology Group) is under active consideration. The focus of InPOG should be cost-effective and logistically feasible protocols for Indian children rather than blindly imported western protocols. To fund nationwide efforts of InPOG, a childhood cancer foundation or “alliance of the stakeholders” comprising parents, volunteers, physicians, and other health professionals is urgently needed. International collaborations to facilitate training of personnel, exchange of knowledge and development of local clinical and laboratory research units will play a vital role in the evolution of InPOG.

Finally, in order to foster pediatric oncology education, postgraduate students should be exposed to the exciting and gratifying aspects of this field. This could, for example, happen through short-term rotation in a PCU. The INTPPO program should evolve into a 3-month structured training program at good centers from a 2-day workshop module at present. Lastly, Promote-Pediatric should focus on large scale national coverage of primary care providers through Indian Medical Association and Indian Academy of Pediatrics for maximum impact.

In conclusion, pediatric oncology in India needs a concerted, collaborative and multidimensional effort to reach international standards. This is also important in order to meet its obligation to ensure the fundamental right of each child to receive good quality health care and chance of cure as stated below by Ponte di Legno group on the right of children with leukemia: “All subscribers to this memorandum, representing the majority of the Childhood Leukemia Treatment Consortia, herewith emphasize the right of all children in the world to full access to the essential treatment of ALL and other cancers, and call upon all authorities concerned to recognize and support all measures that promote this.”[22] Lastly, all of us including physicians, researchers, epidemiologists, administrators, support groups, and all individuals dedicated to the effective treatment of childhood cancer in India should strive to fulfill the dream of legendry Danny Thomas, the founder of St. Jude Children’s Research Hospital that.

“No child should die in the dawn of life”
